# Supplementary material for: Soft Tissue and Biomolecular Preservation in Vertebrate Fossils from Glauconitic, Shallow Marine Sediments of the Hornerstown Formation, Edelman Fossil Park, New Jersey
Source: Biology (Basel). 2022 Aug 2;11(8):1161. doi: 10.3390/biology11081161 (PMC9405258; doi:10.3390/biology11081161)
Supplement: Supplementary file 1 [file biology-11-01161-s001.zip › biology-1800303-supplementary.pdf]

biology

MDPI

Soft tissue and biomolecular preservation in vertebrate fossils from glauconitic, shallow marine sediments of the Hornerstown Formation, Edelman Fossil Park, New Jersey

Kristyn K. Voegele[1], Zachary M. Boles[1,2], Paul V. Ullmann[1], Elena R. Schroeter[3], Wenxia Zheng[3], and Kenneth J. Lacovara[1,2]

1. Department of Geology, Rowan University, Glassboro, NJ
2. Jean and Ric Edelman Fossil Park at Rowan University, Rowan University, Mantua Township, NJ
3. Department of Biological Sciences, North Carolina State University, Raleigh, NC

Keywords: soft tissues; molecular preservation; collagen; Hornerstown Formation; shallow marine; glauconite

1. Supplemental Methods

1.1. Extant sample treatment

These skeletal elements were defleshed and then degreased in a 10% Shout® degreasing solution for 2–3 days on a rocker with 1–2 changes per day. Any remaining soft tissues were removed with a scalpel. The bones were then either studied immediately or stored at -20°C until analysis.

1.2. Protein extraction

For additional details regarding this protocol, see Ullmann et al. [20]. One gram each of bone and sediment were ground into fine powder with separate mortars and pestles. Along with a buffer/"blank" control of only the extraction solutions, each was separately incubated overnight with 0.6M HCl on a shaker at 4°C. After centrifugation, the supernatant was collected (called the 'HCl fraction'), the sample pellet was washed before adding 0.05M ammonium bicarbonate (AMBIC) and being incubated overnight at 65°C on a shaker. Samples were then centrifuged and the supernatant collected (called the 'AMBIC fraction'). HCl fractions were then centrifuged before being precipitated with trichloroacetic acid (TCA) by an overnight incubation at 4°C. For protein precipitation, samples were centrifuged, washed with acetone, decanted, and the HCl fraction pellets were dried in a laminar flow hood at room temperature. The AMBIC fractions were dried in a speed vacuum.

1.3. Polyacrylamide gel electrophoresis (PAGE) with silver-staining

More details of this protocol can be found in Zheng and Schweitzer [51] and Ullmann et al. [20]. Similar to Ullmann et al. [20], we used 50 mg of pre-extracted bone from the AMBIC fraction samples per lane and solubilized samples in 1X Phosphate Buffered Saline (PBS) and combined with an equal volume of 2x Laemmli buffer with 0.01 M dithiothreitol (DTT). As in Ullmann et al. [20], the entire pellet from the fossil sample did not dissolve during this first solubilization and the fossil remaining pellet sample was resuspended and loaded into an additional lane. With this resuspension of the remaining fossil pellet, the majority of it dissolved into solution. All samples (fossil suspensions, sediment, and extraction blanks) were then denatured, centrifuged, and run through a 15% polyacrylamide running gels and 5% stacking gels (made according to standard procedures) for approximately 1 hr at 50 mA. Gels were then incubated in a 50% methanol fixing

solution, washed, and imaged between two 3M™ transparency sheets on a Cannon MX300 scanner to visualize any 'pre-staining' coloration of the gel due to extract sample solution colors. Gels were then transferred back to a tray, sensitized with 0.02% sodium thiosulfate, and incubated with 0.1% silver nitrate. After washing, gels were developed in multiple changes of 0.04% formalin in 2% sodium carbonate until organics became visible and then development was terminated with 5% acetic acid. Pre-staining and post-development images were compared to detect a positive result. An extant control, consisting of 20 µg of pre-extracted extant *Alligator* bone per lane, was conducted in a separate lab at NSCU. Because this modern sample did not visually color the gel prior to treatment with silver nitrate, no pre-staining images were taken.

1.4. Enzyme-linked immunosorbant assay (ELISA)

Following Ullmann et al. [20], we resuspended AMBIC samples in 1X PBS at a concentration of 200 mg of pre-extracted bone per well. Samples were centrifuged to remove particulates, plated, and incubated for 4 hr at room temperature to bind antigens. Following this and each subsequent step, the solution was discarded to remove any unbound antigens or antibodies before proceeding. Non-specific binding was blocked with ELISA blocking buffer (5% bovine serum albumin diluted in 1X PBS with Tween 20 and 2% Thimersol; made following Zheng and Schweitzer [51]) for an additional 4 hr incubation at room temperature. Then, rabbit anti-alligator purified skin collagen antibody (Lot#: AB1432-1, Host#: BSYN 6959, made by Bio-Synthesis) was diluted to a concentration of 1:400 in 1X PBS, and added to a subset of wells; blocking buffer was added to the remaining wells. The plate was incubated overnight at 4°C, then washed with ELISA wash buffer (10x PBS (0.1M) diluted to 1x PBS (0.01M) in Epure water with Tween 20). Next, the plate was incubated for 2 hr with secondary antibodies (alkaline phosphatase-conjugated goat anti-rabbit IgG [H+L]) diluted at 1:2000 in 1X PBS (rather than ELISA blocking buffer, as in Ullmann et al. [20]). The plate was then washed before adding the substrate (one tablet of p-nitrophenylphosphate in 10 mL of 9.8% diethanolamine with 0.5 mM $MgCl_2$) before reading the absorbance. Absorbance was read at timepoints of up to 4 hrs using a Molecular Devices Spectra Max Plus microplate reader (for ancient samples) or a Molecular Devices THERMOmax (for extant samples). Data were acquired in Softmax Pro 4.8. A modern control was also completed with fresh *Alligator* extract loaded at 0.05 µg/well, the same reagents, again using the same protocol in a separate lab at NCSU.

1.5. Immunofluorescence

Our protocols follow Schweitzer et al. [4,15] which are detailed in Zheng and Schweitzer [51]. Fossil demineralization products from the 0.2M HCl treated sample of RU-EFP-00006-11 were collected and washed with Epure water and 1X PBS, before being dehydrated with 70% ethanol. Samples were incubated with a 2:1 solution of L.R. White™ resin/70% ethanol followed by incubation with undiluted L.R. White™ resin. Demineralization products were then transferred to a 0.95 mL gelatin pill capsule (size "00"; 8.81 mm diameter) filled with L.R. White™ resin. The final capsule was polymerized at 60°C for 1–2 days. The fragments of the minced demineralized *Alligator* bone demineralization products were fixed in 10% formalin for 1 hr at room temperature and rinsed before following the above process.

Once polymerized, 200 nm sections were cut (with dedicated separate diamond knives for extant and ancient samples) using a Leica EM UC6 ultramicrotome. Sections were transferred to six-well, Teflon-printed slides (Electron Microscopy Services) and gently heated to dry overnight. All incubations were conducted in a humidity chamber to prevent evaporation of solutions. The modern *Alligator* sample was then treated with 25 µg/ml proteinase K. All wells (see below for procedural changes pertaining to specificity controls) were treated with 0.5M EDTA for 15 min at room temperature for antigen retrieval, then washed IHC-PBS (made following Zheng and Schweitzer [51]). Next, all wells

were treated twice at room temperature with 1 mg/mL sodium borohydride to quench autoflourescence, then washed. To inhibit nonspecific binding, all wells were then incubated with 4% normal goat serum (NGS) in IHC-PBS for 2 hr at room temperature. Next rabbit anti-chicken collagen I antibodies (US Biological, C7510-13B) were added at a 1:40 dilution in primary dilution buffer (PDB; made following Zheng and Schweitzer [51]) to selected wells. All other wells received only PDB. Slides were then incubated overnight.

After primary antibody incubation, slides were washed IHC-PBS with 0.5% Tween 20 and then with IHC-PBS. Biotinylated goat anti-rabbit IgG H+L antibodies (Vector) were then added at a dilution of 1:500 in secondary dilution buffer (SDB; made following Zheng and Schweitzer [51]) to all wells and incubated for 2 hr at room temperature. Following washing as before, all wells were incubated with fluorescein avidin D (FITC) diluted 1:1000 in SDB in the dark, and then washed again. Five to 10 μL of VectaShield H-1000 mounting media was then added to each well. A cover slip was applied and slides were then stored in the dark until imaged at 200 ms exposure with a Zeiss Axioskop 2 Plus microscope with a connected Zeiss AxioCam MRC5 camera. The same camera settings were used for all pictures taken from a particular slide, and any brightness or contrast adjustments were made to all images as a unit, using Corel Paint Shop Pro Photo X2 to preserve their equivalency.

As a specificity control, both modern and fossil samples were exposed to Collagenase A (Roche) to test for non-specific binding as this enzyme degrades collagen. A subset of wells were incubated with 1 mg/mL Collagenase A in Delbucco's PBS to degrade any collagen in the sample. By digesting the collagen, epitopes would be removed, thus decreasing or eliminating binding of specific antibodies. Three variations of this incubation were completed for 1, 3, and 6 hr durations with exchanges hourly and then every 20 min for the last hour. Slides were then treated as above, starting with EDTA incubations. A second specificity control was conducted to test for non-specific paratopes in the polyclonal anti-collagen I antibody by incubating the primary antibodies with *Alligator* collagen prior to incubation with the tissue. US Biological primary antibodies against *Alligator* collagen I were incubated with ~5mg/mL of *Alligator* collagen I before applying to select wells in place of the fresh antibodies at the same concentration and followed the same procedure as above. Replicates conducted with inhibited antibodies were completed in the "modern" lab from this step onward. The *Alligator* collagen I binds to with the anti-collagen I antibodies, and thus the antibodies are no longer available to bind to collagen I in the bone tissue samples. If antibody interactions are specific, binding should be decreased in these controls relative to the test samples.

2. Supplemental Discussion

2.1. Polyacrylamide gel electrophoresis (PAGE) with silver-staining

The "smeared" pattern observed in both of our fossil lanes has been recovered in protein extracts from other fossil bones and is suggestive of molecular degradation [4,15,20,50,59,79]. This result is common and expected for fossil samples because proteins are predicted to be fragmented and damaged by autolytic decay and diagenetic processes [50,80,81]. Concentration of staining at high molecular weights in fossil extracts may be a result of diagenetic crosslinking of proteins [15].

It is not unexpected for there to be some staining for the sediment sample because humics will react with silver nitrate [82]. Humic acids, humin, and fulvic acid are aggregates of organics that form from degrading animal and plant matter found in both sediments and bone [74,80,83]. However, binding in the sediment lanes was minimal and, when present, was distinctly banded, mostly at both ends of the molecular range instead of spread throughout the entire lane as in the fossil bone lanes. Extraction and Laemmli buffer lanes did not display staining, indicating that the organics observed in the fossil bone were not laboratory contaminants. This assay alone does not identify if collagen I is present in the organics identified: the other, more specific assays conducted elucidate this

question. Rather this assay does not falsify the possible presence of protein in this sample, which is supported by the additional molecular assays conducted.

Resuspension of the residual bone pellet from our extractions allowed the positive signal after silver-staining to become more clearly identifiable, as this lane exhibited minimal to no coloration prior to the addition of silver nitrate. Thus, loading of residual, resuspended, pellet materials may be a useful future addition when performing electrophoresis of fossil samples either for PAGE with silver-staining or immunoblot assays, and indeed it may be necessary to visualize organics in samples which stain a gel prior to development. The cause of 'pre-staining' in our bone extract supernatant lanes remains uncertain. One plausible explanation is that because the bones derive from a depositional environment rich in iron, this red-brown color might suggest the presence of an iron compound(s) in the fossil bone protein extracts; however, similar staining would then be expected in the sediment control lanes, which was not found (Figure 5). Alternatively, humic substances present within the fossil but not in the surrounding sediments could account for the 'pre-staining' coloration (cf., [20]), as humics are known to commonly co-solubilize with proteins in fossil bone extracts [74].

2.2. Enzyme-Linked Immunosorbant Assay

As predicted, the fossil bone exhibits a lower absorbance value than the modern bone at the same time point (Figure 5). The more concentrated fossil sample exhibited lower signal than the modern sample, but as its value is over twice background, it is above the threshold for positive signal (e.g., [57,58]). The lower absorbance of fossil samples is likely a result of several factors. Fossil extracts produce smaller protein yields per gram of bone than extracts of extant bone (e.g., [4,59]). Also, the fossil samples have had more time for some of the original protein to decay or epitopes to become damaged (although the positive signal indicates that not all the protein has decayed nor were all epitopes damaged). Diagenetic processes that may have contributed to the preservation of the original fossil protein (e.g., cross-links formed via Maillard reactions [39,80,84]) may also have rendered some epitopes inaccessible. This is supported by our immunofluorescence results, which included an initial increase in fluorescence following brief incubation with digestive enzymes (collagenase) which cleave crosslinks [73].

2.3. Immunofluorescence

The decrease in signal exhibited by modern and fossil tissues exposed to collagenase A identifies: 1) that collagen is present in each sample, and; 2) the primary antibody is binding specifically to collagen [77]. Short incubations (1 hr) of fossil samples with collagenase increased fluorescence, whereas after longer incubations (3 hr and 6 hr) fluorescence decreased. This initial increase in signal has been seen with other fossil samples [20,59] and may result from breakdown of crosslinked protein aggregates formed as byproducts of advanced glycation, lipoxidation, Maillard reactions, and/or Amadori rearrangements (e.g., [10,39,80,84]) which exposes epitopes which were otherwise blocked [73].

3. Supplemental Figure

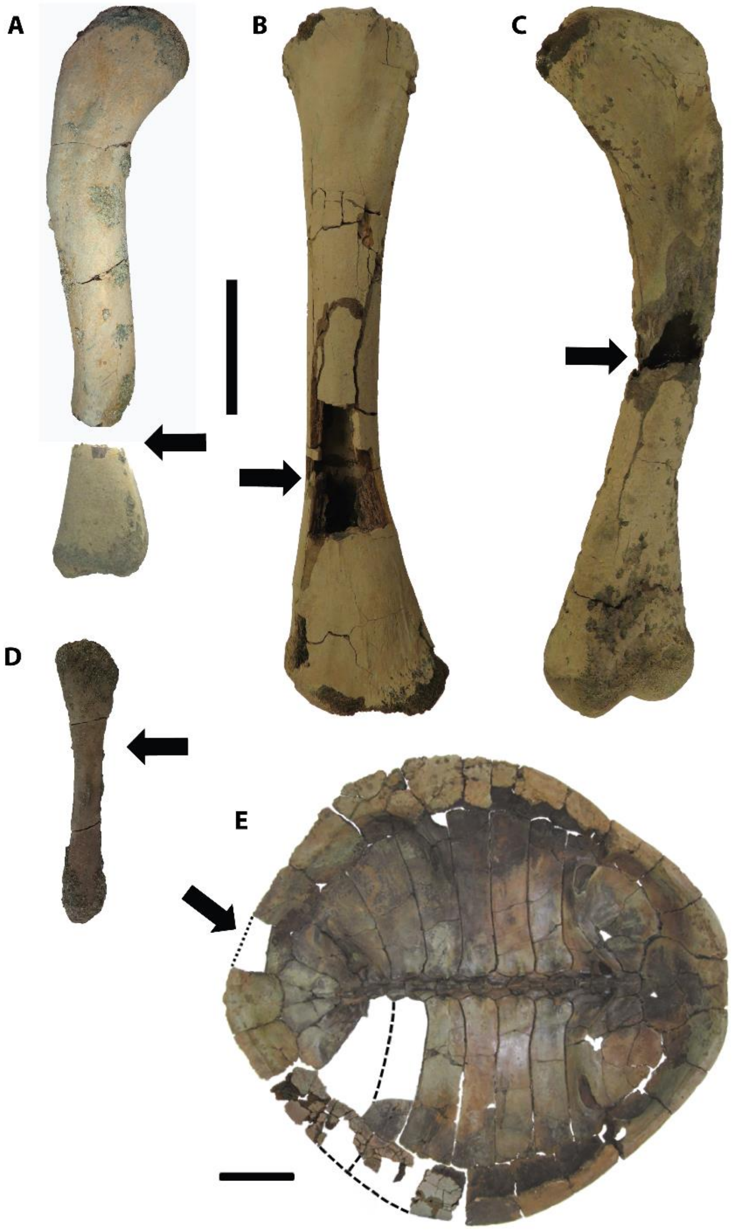

Figure S1. Examples of the well-preserved gross morphology of the specimens examined herein. A) Right femur in ventral view of the crocodile *Thoracosaurus neocesariensis* (RU-EFP-00006-11). B) Left tibia in view of Crocodylia indet. (RU-EFP-00030-1). C) Left humerus in ventral view of Crocodylia indet. (RU-EFP-00030-2). D) Zeugopodial of Testudines indet. (RU-EFP-04161-8). E) Ventral view of a carapace of the turtle *Taphrosphys sulcatus* (RU-EFP-00002-2). Black arrows indicate region of sampling. The majority of cracks and damage seen in these specimens are due to sample collection. Vertical scale bar = 5 cm for A-D. Horizontal scale bar = 10 cm for E.

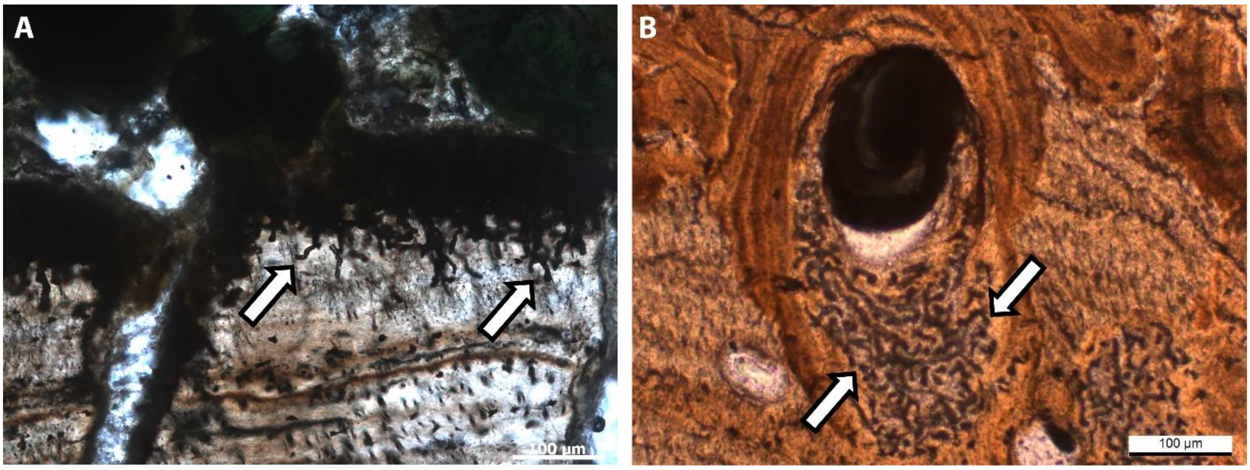

Figure S2. Examples of microbial bioerosion seen in histological thin sections. A) Wedl tunnels in *Thoracosaurus* femur specimen RU-EFP-00006-11. B) Possible microscopical focal destructions in Crocodylia indet. tibia specimen RU-EFP-00030-1. Scale bars as indicated.

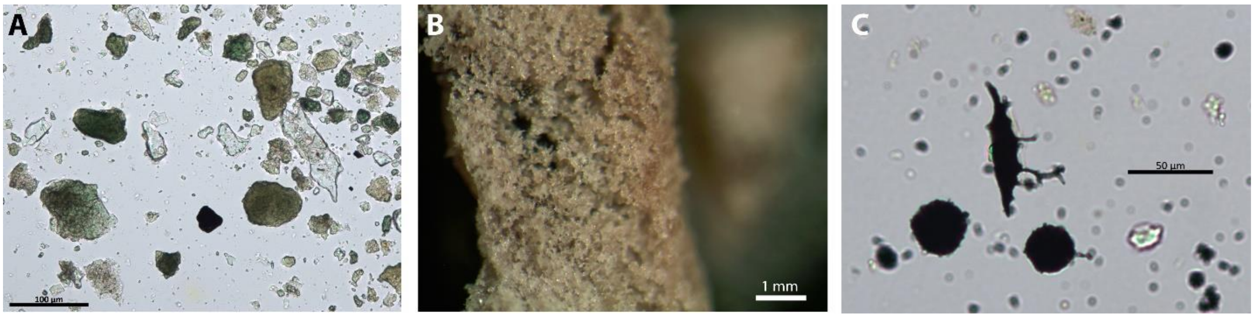

Figure S3. Additional demineralization images. A) Sediment sample with glauconite (green) and quartz (clear) grains visible. B) Close-up of granular and crumbly texture produced by demineralization with HCl (*Thoracosaurus* femur specimen RU-EFP-00006-11). C) A black osteocyte from zeugopodial of Testudines indet. (RU-EFP-04161-8). Scale bars as indicated.

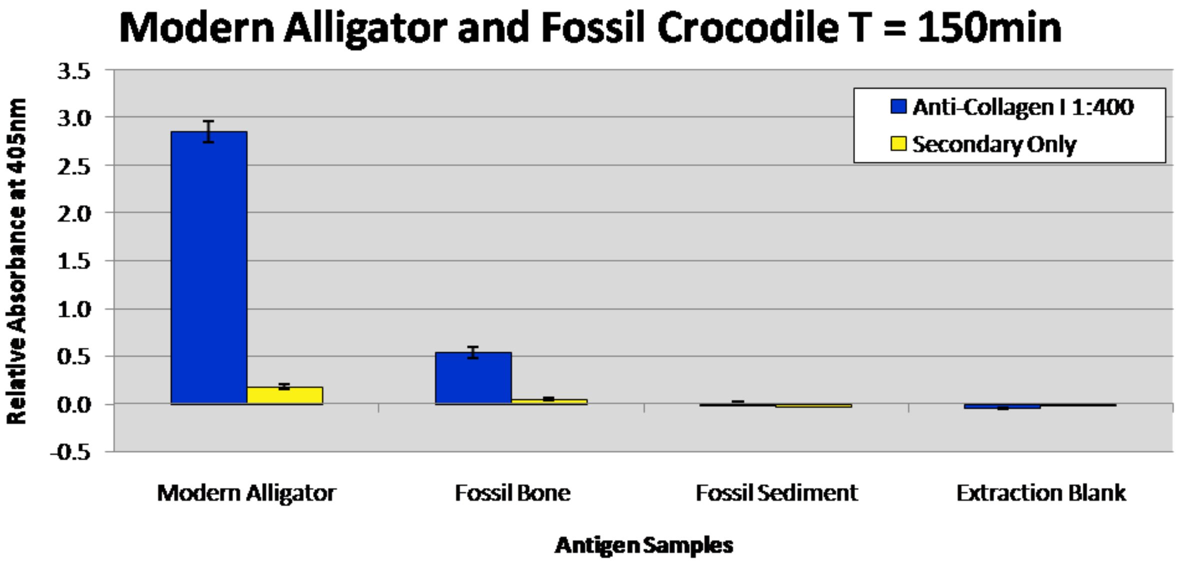

Figure S4. Comparison of enzyme-linked immunosorbant assay results of fossil bone, sediment, and modern *Alligator* chemical extracts. Extraction buffers were also run as a negative control. Fossil and sediment samples were loaded at 200 mg of pre-extracted weight and modern bone was loaded at 0.05µg/well. Blue columns represent absorbance at 405 nm at 150 min, with incubation in anti-chicken collagen I antibodies at a concentration of 1:400. Yellow columns represent the absorbance

of the secondary-only controls for each sample. Although the fossil absorbance is less than that of the modern *Alligator*, its signal is over three times its secondary control, indicating a clear positive signal. Sediment and the extraction blank exhibit low absorbance, indicating they are not a source for the positive signal in the fossil sample. Error bars represent one standard deviation about the mean absorbance of each sample.
